# Transcriptome and metabolome analyses revealed the response mechanism of pepper roots to *Phytophthora capsici* infection

**DOI:** 10.1186/s12864-023-09713-7

**Published:** 2023-10-20

**Authors:** Gang Lei, Kun-Hua Zhou, Xue-Jun Chen, Yue-Qin Huang, Xin-Jie Yuan, Ge-Ge Li, Yuan-Yuan Xie, Rong Fang

**Affiliations:** Institute of Vegetables and Flowers, Jiangxi Academy of Agricultural Sciences, Nanchang, 330200 China

**Keywords:** *Capsicum annuum*, *Phytophthora capsici*, Transcriptome, Metabolome, Salicylic acid, Ca^2+^, Flavonoid biosynthesis pathways

## Abstract

**Background:**

*Phytophthora* root rot caused by the oomycete *Phytophthora capsici* is the most devastating disease in pepper production worldwide, and current management strategies have not been effective in preventing this disease. Therefore, the use of resistant varieties was regarded as an important part of disease management of *P. capsici*. However, our knowledge of the molecular mechanisms underlying the defense response of pepper roots to *P*. *capsici* infection is limited.

**Methods:**

A comprehensive transcriptome and metabolome approaches were used to dissect the molecular response of pepper to *P*. *capsici* infection in the resistant genotype A204 and the susceptible genotype A198 at 0, 24 and 48 hours post-inoculation (hpi).

**Results:**

More genes and metabolites were induced at 24 hpi in A204 than A198, suggesting the prompt activation of defense responses in the resistant genotype, which can attribute two proteases, subtilisin-like protease and xylem cysteine proteinase 1, involved in pathogen recognition and signal transduction in A204. Further analysis indicated that the resistant genotype responded to *P*. *capsici* with fine regulation by the Ca^2+^- and salicylic acid-mediated signaling pathways, and then activation of downstream defense responses, including cell wall reinforcement and defense-related genes expression and metabolites accumulation. Among them, differentially expressed genes and differentially accumulated metabolites involved in the flavonoid biosynthesis pathways were uniquely activated in the resistant genotype A204 at 24 hpi, indicating a significant role of the flavonoid biosynthesis pathways in pepper resistance to *P*. *capsici*.

**Conclusion:**

The candidate transcripts may provide genetic resources that may be useful in the improvement of *Phytophthora* root rot-resistant characters of pepper. In addition, the model proposed in this study provides new insight into the defense response against *P. capsici* in pepper, and enhance our current understanding of the interaction of pepper–*P. capsici*.

**Supplementary Information:**

The online version contains supplementary material available at 10.1186/s12864-023-09713-7.

## Background

*Phytophthora* root and collar rot, caused by the oomycete *P. capsici*, is the most serious disease in pepper production [1, 2], and causes more than $100 million in losses worldwide annually [3]. *Phytophthora capsici*, a soil-born pathogen, can infect almost all parts of the pepper plant and causes different disease symptoms, including root and collar rot and stem, leaf, and fruit blight [4]. Root rot is associated with root darkening and small lesions that can quickly expand to the girdle, and results in the root death [5]. High temperatures and humidity are conducive to the spread of *P*. *capsici* [4]. As a result, this pepper disease is especially severe in southeast China, which is rainy and hot. Unfortunately, current management strategies, including cultural practices, chemical applications, and the planting of resistant hosts, have not been effective in preventing this disease [5]. Therefore, the study of the mechanisms of resistance to *P*. *capsici* is crucial for pepper management improvement and resistance breeding programs [6].

It is well known that diseases occur when pathogens successfully defeat the plant immune system [7]. Plant–pathogen interactions are a dynamic process in which pathogens divert nutrients from hosts for survival and reproduction, and in turn, host plants employ various defense strategies to inhibit pathogen growth [8]. During infection, pathogens secrete effectors as biological tools to invade and propagate in host plants through targeting hosts’ physical barriers for disruption and creating conditions conducive to invasion, and to disturb host cell physiological activity and manipulate plant downstream immune responses [9]. Plants have evolved multi-level resistance mechanisms to defend themselves against pathogen infection, and the activation time and strength of the defense response determine the resistance level of the plant [10]. When plants are attacked by pathogens, a pathogen-associated molecular pattern (PAMP), which is recognized by the cell-surface-localized pattern-recognition receptor (PRR)-triggered immunity (PTI), quickly initiates the first line of defense through calcium (Ca^2+^) influx, ROS production, and mitogen-activated protein kinase (MAPK) activation [10]. During this process, papain-like cysteine proteases (PLCPs) may release PAMPs that are recognized by receptors, activating signaling cascades [11]. Then, subtilisin-like proteases (SBTs), as receptor located in apoplasts, activate downstream immune signaling processes [12, 13], which can act as PRRs in PAMP recognition and immune priming. Pathogen recognition by SBTs is often accompanied by hypersensitive response/ programmed cell death (HR/PCD) to inhibit pathogen growth [14, 15]. The downstream salicylic acid (SA) or jasmonic acid (JA)/ ethylene (ET) signaling pathway is then activated, which can quickly initiate the plant defense response that acts in a positive manner in response to pathogens, such as through the activation of detoxification enzymes, antimicrobial proteins, pathogenesis-related protein (PR), cell wall reinforcement [16, 17], and plant defense secondary metabolites [18]. Leucine-rich repeat (LRR) proteins recognize pathogen effectors and initiate the second line of defense, effector-triggered immunity (ETI) [19]. Moreover, LRR proteins also act as special substrates for the proteolysis of SBTs [20], which may imply a signal transformation and transmission mechanism from SBTs to LRR proteins. ETI also activates HR/PCD and downstream plant hormone-mediated response pathways, such as SA-mediated systemic acquired resistance (SAR) and JA/ET-mediated induced systemic resistance (ISR), to survive and maintain growth [21, 22].

In previous studies, many genes involved in defense responses to *P*. *capsici* have been identified. The chitinase protein genes (*CaChi*) and polygalacturonase-inhibiting protein gene (*CaPGIP1*) can directly inhibit the growth of *P*. *capsici* [23, 24] and provide resistance against *P*. *capsici* by reducing the accumulation of reactive oxygen species (ROS) and triggering the HR and the upregulation of defense-related genes [24, 25]. The ethylene-responsive factor genes *CaAP2/ERF064* and *CaPTI1* are responsible for triggering cell death and involved in the JA/ET signaling pathway to regulate the expression of *CaBPR1*, *CaPR1*, *CaDEF1*, and *CaSAR82* [26, 27]. However, pepper plants with silenced SBP-box family genes (*CaSBP08*, *CaSBP11*, and *CaSBP12*) show enhanced resistance to *P*. *capsici*, along with strongly induced defense genes and decreased cellular damage, indicating negative regulation of the defense response against *P*. *capsici* infection [28–30]. While previous studies have improved the understanding of the molecular mechanisms of pepper in response to *P*. *capsici*, plant responses to pathogen infection are associated with large-scale changes in gene expression and metabolism [31]. Transcriptome technology provides a useful tool to identify genes that might contribute to plant resistance. In a recent study, transcriptome changes analysis of pepper after pathogen infection revealed an important role for the phenylpropanoid biosynthesis pathway in pepper root resistance against *P*. *capsici* [2]. Moreover, plants have different strategies involving the modification of gene expression, activation of several metabolic pathways and post-translational modification of proteins, which culminate into the accumulation of primary and secondary metabolites implicated in plant defense responses [32]. The combination of transcriptomics and metabolomics provides a powerful approach for gaining a deeper and comprehensive insight into the mechanisms of plant defensive responses to pathogen infection at the molecular and cellular levels [33]. For example, terpenoid and flavonoid biosynthesis in cucumber fruit peels associated with age-related resistance to *P*. *capsici* [31], phenylpropanoid metabolism was highly significantly enriched in the resistant *Zanthoxylum bungeanum* following pathogen infection [33], and flavonoid metabolism was observed to play a crucial role in rice resistance to *Meloidogyne graminicola* infection [34]. Therefore, integrated transcriptome and metabolome analysis can offer a unique approach for better understanding of plants in responses to pathogen infection.

*Phytophthora capsici*, as a hemi-biotrophic pathogen, can infect and grow initially as biotrophs but later switch to a necrotrophic phase [35]. At the early stage of infection, the pathogen obtains nutrients from living cells, thus as biotrophs. At this point, in addition to triggering PTI, ETI also plays a role in activating SA-mediated SAR, leading to the accumulation of PR proteins to participate in the defense response against biotrophic pathogens [36, 37]. However, as the pathogen continues to proliferate and cause the death of the host cell, it enters the necrotrophic phase. At this time, the pathogen has completed colonization, and the host plants activate JA/ET-mediated ISR to enhance the expression of defense genes to strengthen defense ability [38, 39].

In the present study, Illumina sequencing technologies were used to compare the transcriptome differences of resistant and susceptible plant roots after *P*. *capsici* infection at three time points, i.e., 0, 24, and 48 hours post-inoculation (hpi). Among them, at 0 hpi, water was inoculated as control; at 24hpi, the infection sites of sensitive plants began to show color changes, which was considered to be the biotrophic phase; and at 48 hpi, the infection sites began to show necrosis, which was considered to be in the necrotrophic phase. Metabolomics technologies based on ultra-high performance liquid chromatography and tandem mass spectrometry (UPLC-MS/MS) platform were employed to analyze changes in the pepper root metabolome at three time points after inoculation. Then, differentially expressed genes (DEGs) and differentially accumulated metabolites (DAMs) were screened to analyze their expression patterns, pathways, and functions in response to *P*. *capsici* infection. The results of this study may further improve our knowledge of the molecular response mechanism of pepper roots to *P*. *capsici* infection and provide a theoretical basis for molecular breeding in developing pepper varieties with resistance to *P*. *capsici*.

## Results

### Phenotypic differences between A198 and A204 in response to *P. capsici* infection

**Fig. 1:**
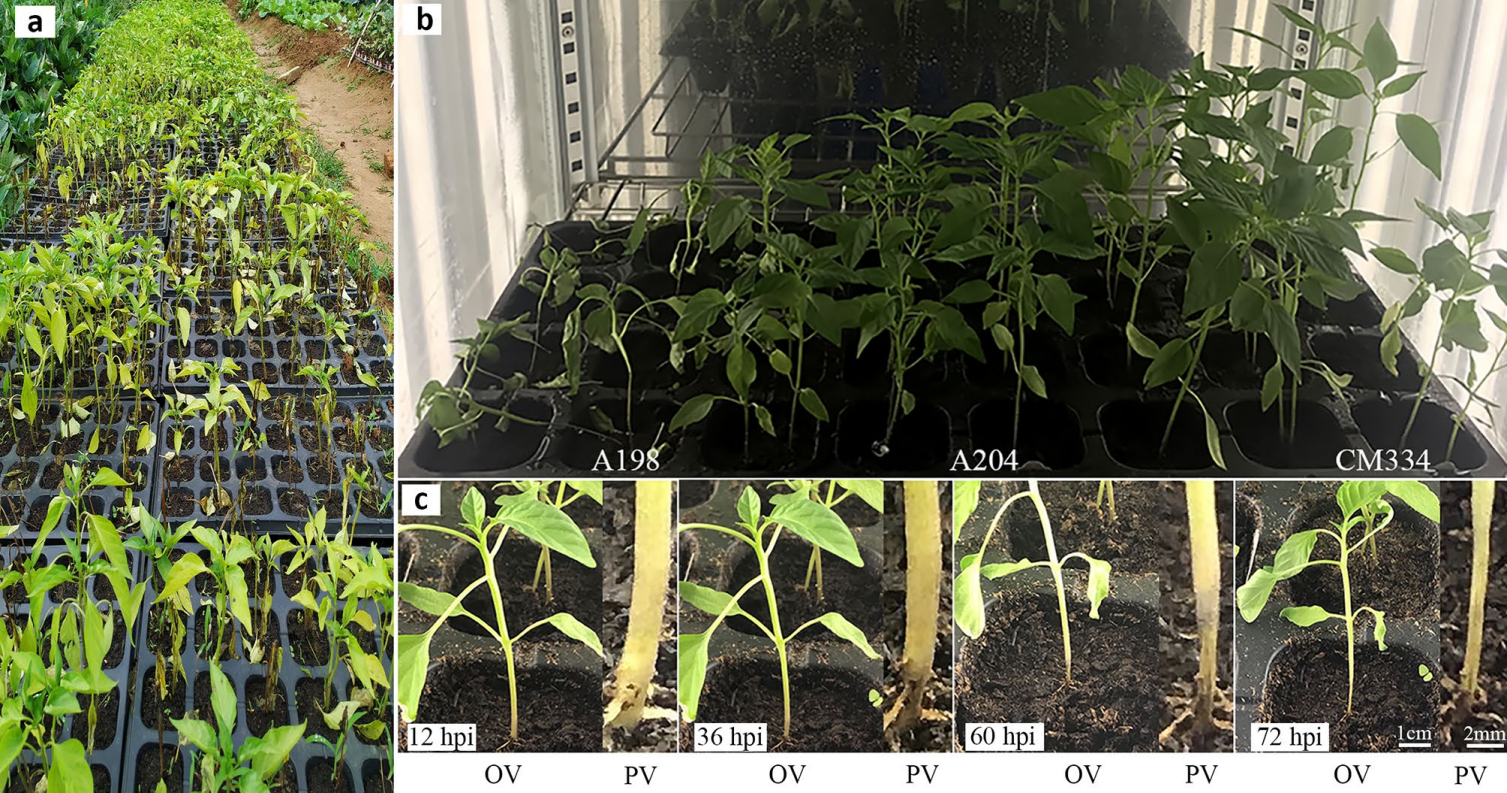
Phenotypic observation of pepper inoculated with *P. capsici*. (**a**) The phenotype of partial pepper germplasm resources 10 days after inoculation for the screening of *Phytophthora* root rot resistance. (**b**) The phenotype of the susceptible genotype A198 and the resistant genotypes A204 and CM334 at 96 h post-inoculation (hpi). (**c**) Symptoms of susceptible genotype A198 at 12, 36, 60, and 72 hpi. OV means the overall view. PV represents the partial view

The two pepper cultivars used in this study were obtained by screening a core collection of 200 pepper germplasm resources for *P*. *capsici* resistance (Fig. 1a). Based on disease phenotypes, this study selected two genotypes with markedly different resistance levels. A204, the resistant genotype, showed the same resistance as the resistant ‘Criollo de Morelos 334’ (CM334) which is one of the most promising sources of resistance to *P*. *capsici* in pepper [40], and had no disease symptoms at 96 hpi with *P*. *capsici* (Fig. 1b). For susceptible genotype A198, all plants were wilted and had a 2–3 cm constriction at the stem base, with defoliation of leaves at 96 hpi (Fig. 1b). To determine the appropriate sampling time points for RNA sequencing (RNA-seq) and metabolomic studies, continuous observation of the symptoms of the susceptible genotype post-inoculation was performed. There were no apparent symptoms at 12 hpi, while brown lesions formed on the junction of the root and stem at 36 hpi, and about 0.5 and 1 cm constrictions were present at the stem base at 60 and 72 hpi, respectively (Fig. 1c). Therefore, 24 hpi was considered a suitable time point for the early stage of infection, which was to be in the biotrophic phase, and 48 hpi, the mid-late stage, was to be in the necrotrophic phase.

### Overview of pepper root transcriptomic responses to ***P. capsici*** infection

Transcriptome analysis was performed on the pepper roots of the two pepper genotypes at 0, 24, and 48 hpi. A total of 118.08 Gb clean data were obtained after removing reads containing adapters, ploy-N, and low-quality reads. The clean data obtained for each sample reached 5.80 Gb, and the Q20 and Q30 base percentage were at least 97.91 and 93.97%, respectively (Table 1). The alignment results showed that at least 88.69% of the high-quality reads were successfully mapped to the reference genome of CM334. Of the mapped reads, 84.71–88.33% were mapped to exon regions, 4.14–5.72% to intron regions, 7.39–9.39% to intergenic regions, and 0.14–0.18% to spliced regions (Supplementary Materials 1:Table S1), which implies that a set number of transcripts were possibly derived from alternative mRNA splicing or new genes. The Pearson’s correlation coefficients (R^2^) between biological replicates were all above 0.99, demonstrating a high degree of biological reproducibility among the samples (Supplementary Materials 7: Fig. S1a). Principal component analysis (PCA) of all transcripts showed that the first two principal components (PCs) explained 74.8% of the total variation, and differences between the genotypes could be clearly displayed, regardless of whether they were infected or not (Supplementary Materials 7: Fig. S1b). Upon *P. capsici* infection, transcriptional profiles changed in opposite directions between the two genotypes, indicating that the two genotypes may resist *P. capsici* infection through different response mechanisms.

**Table 1:** General information of sequencing reads and reads that mapped to the reference genome

Sample	Clean Read	Clean Base (G)	GC content (%)	Q20 (%)	Q30 (%)	Mapped reads
S0-1	45,238,630	6.75	42.91	97.99	94.14	42,533,284 (94.02%)
S0-2	43,915,334	6.55	42.93	98.12	94.50	41,338,562 (94.13%)
S0-3	41,189,220	6.15	42.87	97.91	93.97	38,679,114 (93.91%)
S1-1	41,592,546	6.20	43.04	97.99	94.16	38,598,001 (92.80%)
S1-2	41,453,026	6.18	42.96	97.93	93.98	38,548,999 (92.99%)
S1-3	47,701,382	7.12	43.03	98.04	94.28	44,212,033 (92.69%)
S2-1	44,443,902	6.64	43.42	98.09	94.37	39,637,240 (89.18%)
S2-2	44,636,102	6.66	43.39	98.06	94.27	39,908,893 (89.41%)
S2-3	45,383,130	6.77	43.53	98.06	94.30	40,512,719 (89.27%)
R0-1	48,827,806	7.27	43.15	98.05	94.34	45,816,990 (93.83%)
R0-2	38,843,400	5.80	43.04	97.95	94.18	36,478,638 (93.91%)
R0-3	50,413,708	7.53	43.07	98.07	94.45	47,289,383 (93.80%)
R1-1	39,227,692	5.85	42.89	98.07	94.44	36,438,894 (92.89%)
R1-2	42,358,224	6.32	43.56	97.95	94.16	37,566,792 (88.69%)
R1-3	48,981,512	7.32	42.86	97.98	94.17	45,834,779 (93.58%)
R2-1	40,608,484	6.06	42.86	97.94	94.04	38,083,232 (93.78%)
R2-2	44,990,206	6.71	42.88	98.00	94.10	42,170,748 (93.73%)
R2-3	41,544,724	6.20	42.85	98.05	94.27	38,930,876 (93.71%)

Note: R and S represent the resistant (A204) and susceptible (A198) genotype, respectively. R0, R1, and R2 (S0, S1, and S2) indicate 0, 24 and 48 h post inoculation (hpi) of the resistant genotype A204 (the susceptible genotype A198). The numbers (1, 2, and 3) at the end represent three biological replicates

### Functional annotation and identification of DEGs

**Fig. 2:**
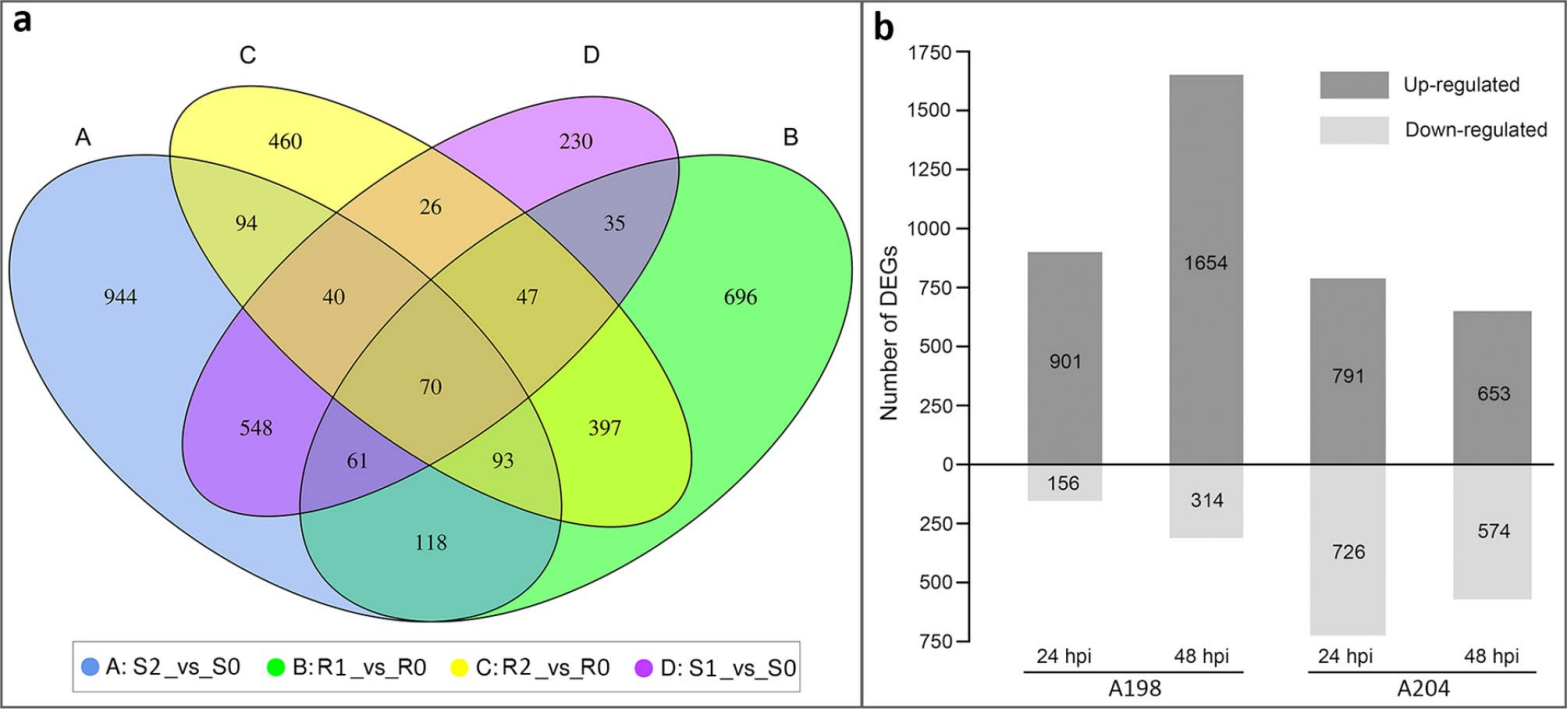
Transcriptional changes upon *P. capsici* infection in A198 and A204. (**a**) Venn diagram of differentially expressed genes (DEGs). R1/ R2 vs. R0 represent a comparison between 24/ 48 h post-inoculation (hpi) and 0 hpi, in the resistant genotype. S1/ S2 vs. S0 represent a comparison between 24/ 48 hpi and 0 hpi in the susceptible genotype. (**b**) Number of up- and downregulated DEGs of two genotypes at 24 and 48 hpi

Through database search of detected transcripts, 35,642 genes (including 4750 new genes) were annotated, of which 9546, 25,863, 23,776, 17,636, 25,893, 21,600, 29,437, and 35,576 genes were annotated in the Cluster of Orthologous Group (COG), Gene Ontology (GO), KEGG, Karyotic Ortholog Groups (KOG), Pfam, Swiss-Prot, evolutionary genealogy of genes: Non-supervised Orthologous Groups (eggNOG), and non-redundant (NR) databases, respectively. To identify DEGs, pairwise comparisons were independently performed between each time point during infection and at 0 hpi in both genotypes. A total of 1517 and 1227 DEGs were identified in the resistant genotype, and 1057 and 1968 DEGs were determined in susceptible genotype A198 at 24 and 48 hpi, respectively (Fig. 2; Supplementary Materials 2: Table S2). The DEGs from the same genotype overlapped more, but the DEGs from different genotypes were mostly unique (Fig. 2a). Among the DEGs, the number of upregulated DEGs was higher than that of downregulated DEGs, and the downregulated DEGs in A204 were far more numerous than those in A198 at all time points (Fig. 2b). Additionally, a hierarchical cluster analysis of all DEGs was performed to assess the reproducibility of RNA-sequencing data, and the cluster heatmap showed that the three biological replicates of each sample group were clustered into one cluster, which indicated the reliable reproducibility of RNA-sequencing (Supplementary Materials 7: Fig. S2).

### DEGs enrichment analysis

**Fig. 3:**
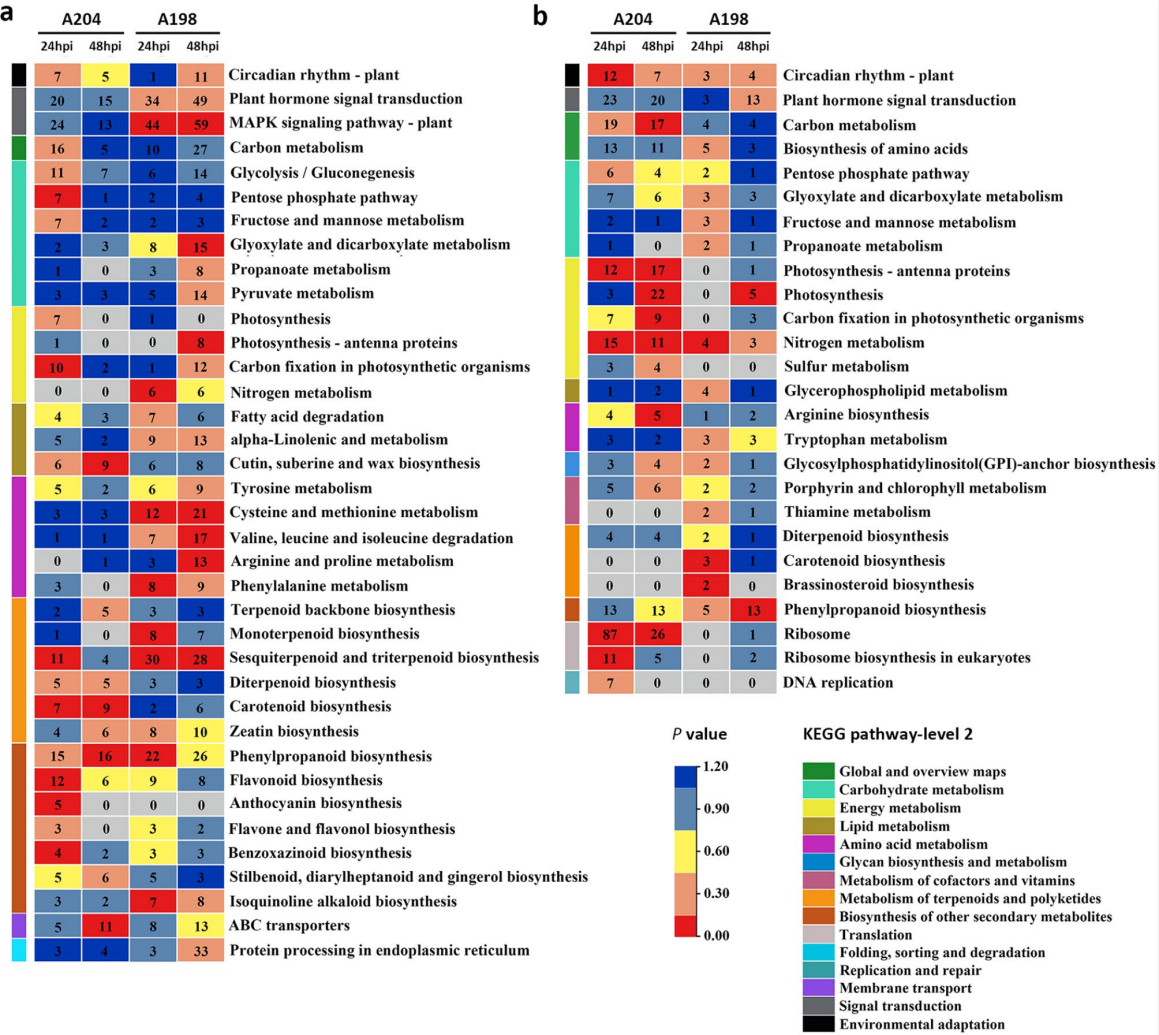
Kyoto Encyclopedia of Genes and Genomes (KEGG) pathway enrichment analysis of up- (**a**) and downregulated (**b**) differentially expressed genes (DEGs). The number in the box is the number of DEGs enriched in that pathway per sample

To determine the pathways of the DEGs involved in pepper response to *P*. *capsici* infection, a KEGG enrichment analysis of the DEGs was performed. A total of 37 and 26 KEGG pathway categories were enriched in the up- and downregulated DEGs, respectively (Fig. 3). Among them, the 13 enriched KEGG pathways shared by both up- and downregulated DEGs mainly involved carbohydrate metabolism, energy metabolism, signal transduction, and the biosynthesis of other secondary metabolites. Correspondingly, 24 and 12 KEGG pathway categories were uniquely enriched in up- and downregulated DEGs, respectively (Fig. 3). For signal transduction, the “plant hormone signal transduction pathway” and “MAPK signaling pathway” were significantly enriched in the susceptible genotype at 24 and 48 hpi among the upregulated DEGs. The number of DEGs involved in the two pathway categories showed an increasing trend from 24 to 48 hpi in the susceptible genotype, while the opposite trend was found in the resistant genotype. These results suggest that the resistant genotype may respond to *P*. *capsici* earlier than the susceptible genotype. Notably, most pathways involved in the “metabolism of terpenoids and polyketides” and “biosynthesis of other secondary metabolites” were significantly enriched in the resistant genotype at 24 hpi, including “sesquiterpenoid and triterpenoid biosynthesis”, “diterpenoid biosynthesis”, “carotenoid biosynthesis”, “phenylpropanoid biosynthesis”, “flavonoid biosynthesis”, “anthocyanin biosynthesis”, and “flavonoid and flavonol biosynthesis”, which were considered to be related to the response to biotic and abiotic stress. In terms energy metabolism, namely “photosynthesis-antenna proteins”, “carbon fixation in photosynthetic organisms”, and “nitrogen metabolism” were significantly enriched in the susceptible genotype in the upregulated DEG group, while these pathways were significantly enriched in the resistant genotype in the downregulated DEG group. Unexpectedly, most pathways involved in amino acid metabolism, such as “tyrosine metabolism”, “cysteine and methionine metabolism”, “valine, leucine and isoleucine degradation”, “arginine and proline metabolism”, and “phenylalanine metabolism”, were significantly enriched only in the susceptible genotype. The same result was found for the pathways related to “lipid metabolism” and “fatty degradation”. These results may suggest that the enhanced energy metabolism, as well as amino acid and lipid catabolism, was enhanced to meet energy requirements in the susceptible genotype.

### DEGs associated with disease resistance

To better understand the network of DEGs that responded to *P*. *capsici* infection, the transcriptional changes of resistant and susceptible pepper roots were visualized using MapMan software. The heatmap shows a general overview of the differences in the response to *P*. *capsici* infection between the two genotypes (Supplementary Materials 7: Fig. S3). As already highlighted by the DEG analysis, there were more DEGs involved in cell wall strengthening, signaling, and secondary metabolite synthesis at 24 hpi in the resistant genotype than in the susceptible genotype (Supplementary Materials 7: Fig. S3a and b), while at 48 hpi, the opposite response to *P*. *capsici* infection was observed in the DEGs of the two genotypes (Supplementary Materials 7: Fig. S3c and d), indicating that the resistant genotype reacted more quickly to infection than the susceptible genotype.

**Structural defense.** The plant cell wall is the first line of defense, providing the plant with initial defense and signal perception against pathogen attack. In this study, five subtilisin-like protease genes were found to be especially induced in the resistant genotype at 24 hpi (Supplementary Materials 3: Table S3). Interestingly, from 24 to 48 hpi, the number of upregulated DEGs encoding for the LRR protein in the resistant genotype decreased, while the number of DEGs in the susceptible genotype continued to increase. Furthermore, genes responsible for cell wall strengthening, such as *proline-rich protein* (*PRP*), *cellulose synthase* (*CesA*), and *syntaxin*, were also differentially expressed. Unexpectedly, the strong repression of most unigenes encoding proline-rich protein occurred in both genotypes at 24 and 48 hpi, while the comparison of the baseline levels of the detected DEGs between A204 and A198 (R0 vs. S0) showed higher levels of six proline-rich protein gene transcripts (Supplementary Materials 3: Table S3), which may contribute to improved pathogen defense in the resistant genotype. For cellulose synthase, two genes were upregulated in the resistant plants at 48 hpi. Only one gene, *LOC107853789*, was upregulated in the susceptible plants, and this gene had a higher baseline level (25.35-fold) in the resistant genotype (Supplementary Materials 3: Table S3). In addition, the A204 response to *P*. *capsici* was characterized by the earlier induction of cell wall degradation-related enzymes, such as polygalacturonase (PG) and pectinesterase (PE), as well as pectate lyase. At 24 hpi, six DEGs encoding PG were induced in A204, but only one was induced in the susceptible genotype. More DEGs coding for PE and pectate lyase were upregulated at 48 hpi in A198. These results indicate that the degradation of the cell wall, as well as the response to pathogen attack, occurred earlier in the resistant genotype than in the susceptible genotype.

**Signal transduction.** To mount an effective defense, plants rapidly transmit stress signals to trigger various downstream defense mechanisms through different signaling pathways, including calmodulin (CaM) and phytohormone signaling pathways. Nine CaM-related DEGs were upregulated in the resistant genotype (Supplementary Materials 3: Table S3), suggesting a key role of the Ca^2+^-dependent signaling pathway in resistant genotype A204. It is well-known that the SA, JA, and ET pathways play important roles in signal transduction in response to biotic stress. In this regard, three SA-related DEGs encoding ankyrin repeat-containing proteins were induced at 24 hpi in A204. A total of 37 DEGs related to ET-responsive factors were identified. among them, 18 DEGs were expressed in A198 and nine in A204 (Supplementary Materials 3: Table S3). For JA-related genes, three DEGs coding for lipoxygenase (LOX), one for a ZIM domain-containing protein, and one for TIFY were upregulated in the susceptible genotype, but only one DEG encoding TIFY was induced in A204. Furthermore, 11 auxin-related genes were identified in the two genotypes. Interestingly, they were differentially expressed in A198 only at 48 hpi but differentially expressed at 24 and 48 hpi in A204. Similar to the changes in auxin-related DEGs, three abscisic acid (ABA) receptor genes were upregulated in A198 only at 48 hpi. In addition, activation of the MAPK cascade and WRKY transcription factor family members were also observed (Supplementary Materials 7: Fig. S3). *MPKK5* genes were suppressed in both genotypes at 48 hpi. Six and seven *WRKY* genes were induced in A198 at 24 and 48 hpi, respectively (Supplementary Materials 3: Table S3). These results suggest that the two genotypes may transmit stress signals through different signaling pathways.

**Chemical defense.** When a pathogen breaks through the first line of defense, plants usually fight pathogens and repair themselves through the employment of numerous specialized proteins, such as xylem proteinase and pathogenesis-related protein (PR). As shown in Fig. S3 (Supplementary Materials 7), more genes encoding specialized proteins were differentially induced in A204 at 24 hpi, including xylem cysteine proteinase 1 (XCP1), pathogenesis-related protein 1 (PR1), endo-1,3(4)-beta-glucanase (PR2), chitinase (PR3 and PR4), thaumatin-like protein (PR5), and peroxidase (PR9) (Supplementary Materials 3: Table S3). Two XCP1 genes (*LOC107851745* and *LOC107866767*) were uniquely induced in A204. For PR1, two DEGs were upregulated only at 24 hpi in the resistant genotype, while one was upregulated in the susceptible genotype at 48 hpi, suggesting a late defensive response in the susceptible materials. Three PR2-related genes, namely *LOC107866197*, *LOC107839367*, and *newGene_10657*, were upregulated only in A204, while one (newGene_10487) was induced in A198 (Supplementary Materials 3: Table S3). The PR3-, PR4-, and PR5-related DEGs were most induced in the susceptible genotype, especially nine chitinase-related genes that were upregulated at 48 hpi (Supplementary Materials 3: Table S3), which demonstrated that a strong defensive response occurred at that time. In addition, eight genes implicated in biosynthesis of flavonoid were uniquely upregulated in A204 at 24 hpi (Supplementary Materials 3: Table S3).

### Validation of the expression of selected DEGs

**Fig. 4:**
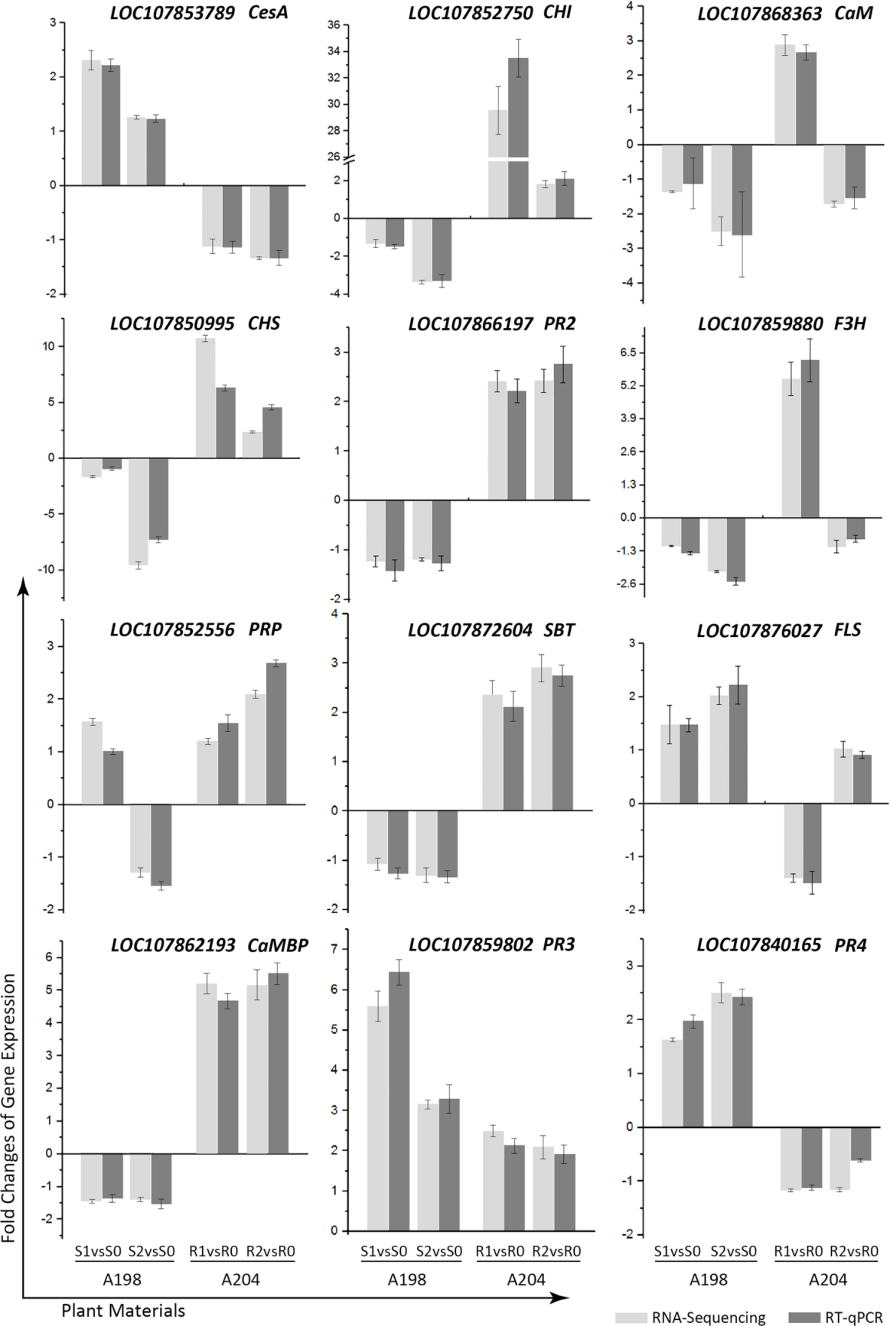
Comparison of real-time quantitative polymerase chain reaction (RT-qPCR) and RNA-Sequencing results. R1/ R2 vs. R0 represent a comparison between 24/ 48 h post-inoculation (hpi) and 0 hpi in the resistant genotype. S1/ S2 vs. S0 represent a comparison between 24/ 48 hpi and 0 hpi in the susceptible genotype

To confirm the validity of the transcriptome data, real-time quantitative polymerase chain reaction (RT-qPCR) analysis was performed on 12 selected genes (Fig. 4). These selected genes were involved in signal transduction (*CaM LOC107868363*, *CaMBP LOC107862193*, and *SBT LOC107872604*), cell wall enhancement (*PRP LOC107852566* and *CesA LOC107853789*), pathogen resistance (*PR2 LOC107866197*, *PR3 LOC107859802*, and *PR4 LOC107840165*), and flavonoid biosynthesis (*CHS LOC107850995*, *CHI LOC107852750*, *F3H LOC107859880*, and *FLS LOC107876027*), which were either directly or indirectly linked to plant resistance. The comparison between the two techniques revealed substantial agreement for all 12 genes differentially expressed upon pathogen infection.

### Metabolite profiles

**Fig. 5:**
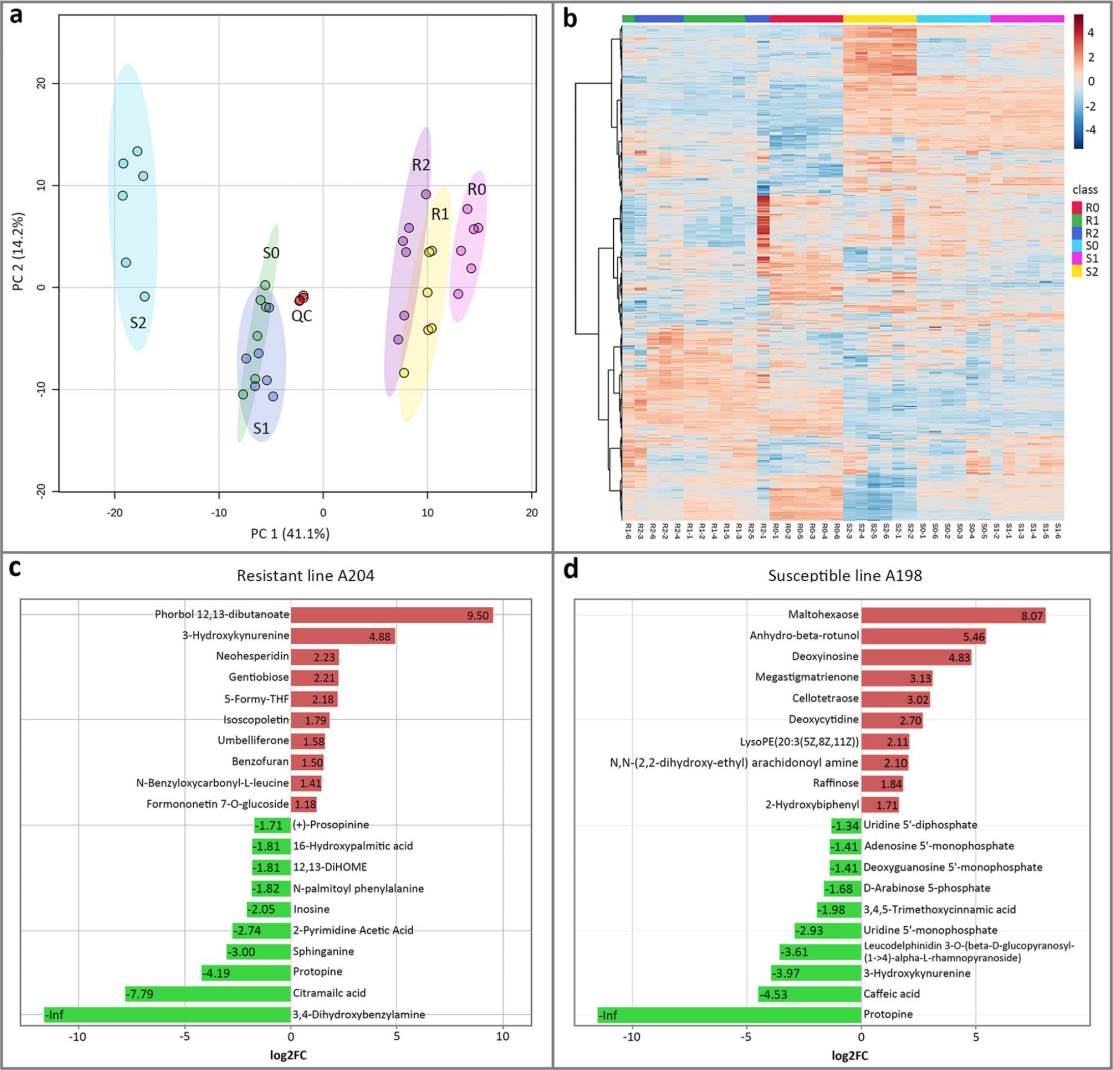
Metabolic changes upon *P. capsici* infection in A198 and A204. (**a**) Principal component analysis (PCA) of all metabolites detected at 0 (R0 and S0), 24 (R1 and S1), and 48 (R2 and S2) hours post-inoculation (hpi) in both genotypes. (**b**) Clustering analysis heat map of the expression of differentially accumulated metabolites (DAMs) detected in each sample. Color coding represents the metabolite concentrations with high (red) and low (blue) expression. Top 10 DAMs in the resistant genotype A204 (**c**) and susceptible genotype A198 (**d**)

Untargeted metabolomics analyses were performed on pepper root extracts using an UPLC-MS/MS platform. After data filtering and identification, 688 compounds (including 514 in positive ion mode and 174 in negative ion mode) were acquired from the root extracts of all samples (Supplementary Materials 4: Table S4). PCA of the metabolome data showed tight clustering of the replicate samples of both genotypes and quality control samples, confirming the reproducibility of the results (Fig. 5a). PCA score plots further revealed that, similar to the transcriptome data, differences between genotypes could be clearly displayed based on PC1, regardless of whether they were infected. A heatmap of all analyzed ions revealed distinct hierarchical clustering of the samples based on genotype, and ions differentially accumulated with either different genotypes or different time points (Fig. 5b). To identify the DAMs, orthogonal partial least squares-discriminant analysis (OPLS-DA) models were constructed to maximize the difference between the experimental sample groups (R1, R2/S1, and S2) and the control sample groups (R0/S0). The OPLS-DA models (Q^2^ > 0.9) showed reliable predictability and significant biochemical perturbation in the experimental groups (Supplementary Materials 7: Fig. S4 and 5).

Metabolites with fold-changes of ≥ 2.0 or ≤ 0.5, VIP values > 1.0, and *p*-values < 0.05 were selected as DAMs. There were 53 and 80 DAMs in the resistant genotype at 24 and 48 hpi, respectively (Supplementary Materials 5: Table S5). The top 10 significantly altered metabolites at 24 hpi are showed in Fig. 5c. Among them, the upregulated DAMs mainly consisted of phenolic compounds, including flavonoids (neohesperidin and formononetin 7-O-glucoside) and phenylpropanoids (isoscopoletin, umbelliferone), and the top 10 downregulated DAMs mainly included lipids (sphinganine, 12,13-DiHOME, and 16-hydroxypalmitic acid). For the susceptible genotype, 32 and 108 DAMs were identified at 24 and 48 hpi, respectively (Supplementary Materials 5: Table S5). The top 10 upregulated DAMs mainly included carbohydrates (maltohexaose, cellotetraose, and raffinose) and nucleotide derivates (deoxyinosine and deoxycytidine), and the downregulated DAMs mostly consisted of nucleotide derivatives and phenylpropanoids (Fig. 5d).

### Association of transcriptomic and metabolomic changes involved in the response of pepper to *P. capsici* infection

**Fig. 6:**
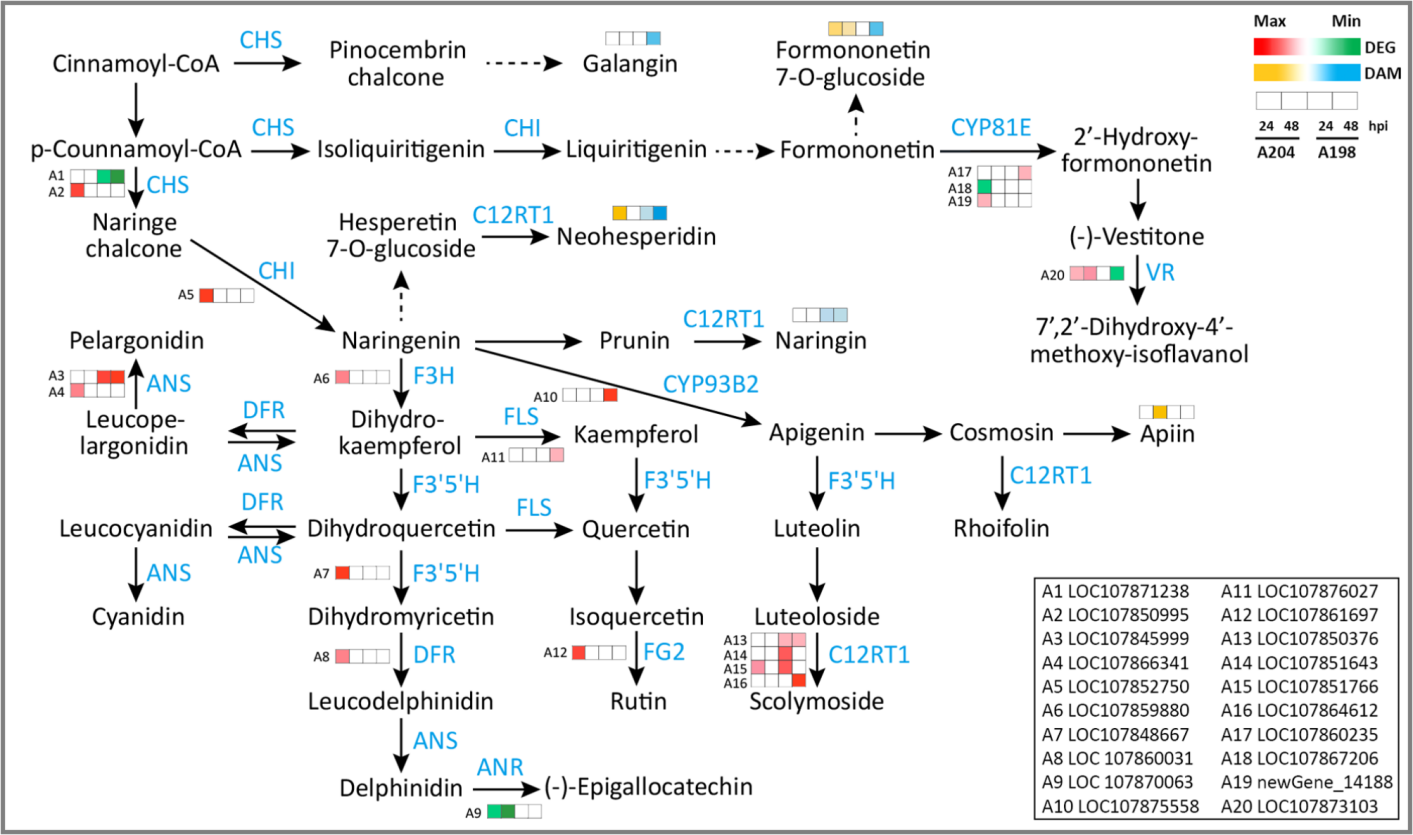
Heatmap of the log2-fold changes in genes and metabolites involved in the flavonoid biosynthesis pathways in both genotypes upon *P. capsici* infection at 24 and 48 h post-inoculation (hpi). In the schemes of cascades, compounds are shown in black font and genes in blue. ANS, anthocyanidin synthase; ANR, anthocyanidin reductase; CHI, chalcone isomerase; CHS, chalcone synthase; C12RT1, flavanone 7-O-glucoside 2’’-O-beta-L-rhamnosyltransferase; CYP81E, isoflavone/4’-methoxyisoflavone 2’-hydroxylase; CYP93B2, flavone synthase II; DFR, dihydroflavonol reductase; F3H, flavanone 3-hydroxylase; F3’5’H, flavonoid 3’,5’-hydroxylase; FG2, flavonol-3-O-glucoside L-rhamnosyltransferase; FLS, flavonol synthase; FNS, flavone synthase; VR, vestitone reductase. The heatmap was drawn based on the KEGG pathway maps [41]

To obtain more information on the physiological changes of pepper in response to *P*. *capsici*, this work concentrated on the connection between gene expression and metabolite changes. Based on the KEGG analysis results of DEGs and DAMs, a heatmap of the flavonoid biosynthesis pathways flow chart was drawn in this study (Fig. 6). The results showed that five flavonoid compounds, namely neohesperidin, naringin, apiin, galangin, and formononetin 7-O-glucoside, were accumulated in the resistant genotype A204, while in the susceptible genotype A198, these DAMs were decreased or unchanged. The expression patterns of most genes were similar to those of the metabolites (Fig. 6). For two *chalcone synthase* (*CHS*) genes, *LOC107850995* was upregulated in A204 at 24 hpi, while the expression of *LOC107871238* was suppressed in A198 at both time points (Fig. 6; Supplementary Materials 3: Table S3). Chalcone isomerase (CHI) catalyzes the cyclization of chalcone into flavanone, and the gene *LOC107852750* encoding CHI was strongly induced only in A204 at 24 hpi (Fig. 6; Supplementary Materials 3: Table S3). As with *CHI*, five genes involved in flavonoid biosynthesis, namely *flavanone 3-hydroxylase* (*F3H*), *flavonoid 3’,5’-hydroxylase* (*F3’5’H*), *dihydroflavonol reductase* (*DFR*), *flavonol-3-O-glucoside L-rhamnosyltransferase* (*FG2*), and *vestitone reductase* (*VR*), were uniquely upregulated in resistant genotype A204 at 24 hpi. These results of combined transcriptome and metabolome analysis suggest that the resistance of genotype A204 could be related to its special ability or earlier ability to regulate gene expression in flavonoid biosynthesis pathways and the accumulation of flavonoids.

## Discussion

### Structural defense in response to *P. capsici* infection in pepper

The cell wall, the first barrier of defense, provides the pepper plants with initial defense and signal perception against pathogen attack though sensing and defense components on the cell wall. In this regard, many genes related to the primary innate immune system were recovered in both genotypes in response to *P*. *capsici* infection. In particular, the majority of DEGs encoding subtilisin-like proteases were upregulated only in A204 at 24 hpi. Plant subtilisin proteases are not only involved in all aspects of the plant life cycle, but also in the response to biotic stress by mounting an effective defense strategy through the activation of signaling cascades and causing direct damage to the pathogen [12, 13]. Likewise, LRR proteins act as membrane-bound signaling molecules to recognize pathogen effectors and activate ETI [19]. In this study, the number of upregulated DEGs coding for LRR proteins decreased from 24 to 48 hpi (22 to 8) in A204 but increased (17 to 23) in A198, which may indicate a late response to pathogen infection in the susceptible genotype. With *P*. *capsici* infection, more DEGs involved in cell wall reinforcement (such as *PRPs* and *CesA*) were differentially changed in A204. PRPs are important components of cell wall proteins that play pivotal roles in cell wall signal transduction cascades and secondary wall formation [16, 42]. Cellulose, a main component of cell walls, is produced by CesA [17]. Cell wall degradation enables pathogen invasion of plant tissues, although it may trigger a plant defense response. In the present study, the data showed the strong expression of DEGs involved in PGs at 24 hpi in A204, and PEs and pectate lyase in the susceptible genotype at 48 hpi, which indicated the successful invasion of *P*. *capsici* in both genotypes and an earlier defense response in the resistant genotype. Similar results have been observed in melon*–Fusarium oxysporum* f. sp. *melonis* Race 1.2 Pathosystem [43].

### Crucial role of Ca^2+^ and SA signaling pathways in pepper against *P. capsici* infection

Timely and effective signal transmission is crucial for activating downstream defense mechanisms in plant resistance against biotic stress. Ca^2+^ is a universal second messenger involved in various cellular processes and acts as the earliest signaling event in plant–pathogen interactions [44, 45]. Ca^2+^ signals can be transduced by the ubiquitous small calcium-binding protein CaM, and its interactions are modulated through the binding of CaM to other proteins, such as calmodulin-binding protein (CaMBP), which participates in the defense response [46, 47]. The data in this study revealed strong expression of genes encoding CaM only in A204. Interestingly, continuous expression of CaMBP-related DEGs was observed in A204 at 24 and 48 hpi. Additionally, CaM-binding activity has been associated with plant defense responses by acting on homeostasis regulation by SA [45], which, as an important plant hormone, participates in defense responses during ETI and microbe-associated molecular pattern (MAMP)-induced immunity in plants [48]. More DEGs encoding ankyrin repeat-containing proteins associated with plant disease resistance mediated by SA [49, 50] were induced in A204 at 24 hpi than in A198 at 24 hpi. Interestingly, one CaM transcription activator factor gene (*LOC107863749*), which is involved in SA biosynthesis and SA-mediated immune responses [51], was upregulated only in the resistant genotype at 24 hpi. These results indicate the key roles of the Ca^2+^ and SA signaling pathways in response to *P*. *capsici* in the resistant genotype, which had been reported in *Arabidopsis–Pseudomonas syringae* pathosystems, where *A. thaliana* signal responsive protein1 (AtSR1), regulated by Ca^2+^/CaM, negatively regulates the SA level through repressing the expression of *enhanced disease susceptibility 1* (*EDS1*) [52].

The JA/ET-mediated pathway is involved in plant disease resistance and is antagonistic to the SA-mediated defense response pathway [10, 53]. The majority of JA- and ET-related DEGs were upregulated in the susceptible genotype at both time points, suggesting that the JA/ET-mediated signaling pathway plays an important role in the defense against *P*. *capsici* in A198, and indicating that *P*. *capsici* has been switching into necrotrophic phase, which is consistent with the observed 0.5 cm constrictions at the stem base of A198 at 60 hpi. ABA is a positive regulator of the defense response through the regulation of stomatal closure at the pre-invasive level, while in contrast, it has a negative effect on disease resistance at the post-invasive level due to the inhibition of defense hormone-triggered resistance [54, 55]. In this study, we found that ABA-related genes were all exclusively upregulated in the susceptible genotype at 48 hpi. Therefore, we speculated that an inefficient defense response exists in the susceptible genotype at the late stage of infection due to interference with the ET signaling pathways caused by ABA.

### Modulation of defense-related proteins and secondary metabolites in pepper

As a pathogen continues to invade, the stress signal is quickly transmitted downstream, leading to the biosynthesis of defense-related factors, such as XCP1, PR proteins, and antibacterial compounds [10, 56, 57]. XCP1, a prominent enzyme in the plant apoplast, belongs to the PLCPs, which can activate defense responses by producing PAMP-like peptides that are recognized by PRRs and by participating in pathogen effector-induced HR [11, 58]. The present study showed that two DEGs coding for XCP1 were uniquely induced in the resistant genotype, suggesting the positive defense response of XCP1 in pepper to *P*. *capsici*. PR proteins are a group of diverse proteins induced by phytopathogens and defense-related signaling molecules [59]. These proteins are key to SAR, an inducible plant immune response that prevents further infection of noninfected parts of the host [59]. In this study, most PR1 and PR2 protein-related genes were upregulated in the resistant genotype at 24 hpi, while genes encoding PR3, PR4, and PR5 proteins were mainly induced in the susceptible genotype. Interestingly, PR1 and PR2 proteins can be activated by the SA pathway, and PR1 is a molecular marker for SA-induced SAR response [59, 60], which is consistent with the dominant role of SA signaling in A204. PR3 and PR4 proteins can be activated by the JA pathway and provide only local acquired resistance [59], which is also consistent with the JA/ET-mediated pathway in A198.

Plants are well known to respond to pathogens through the activation of the phenylpropanoid pathway, leading to the biosynthesis of flavonoids, isoflavonoids, and phenolics [2, 31, 33, 34]. The combined transcriptome and metabolome analysis showed that DEGs and DAMs involved in flavonoid biosynthesis pathways were significantly enriched (Figs. 3 and 6), suggesting that these pathways could be beneficial to improving pepper plant resistance to *P*. *capsici*. The crucial role of the activation of flavonoid biosynthesis pathways in response to pathogens has been reported in cucumber [31], *Zanthoxylum bungeanum* [33], and rice [34].

### Proposed model of *P. capsici* resistance mechanisms in pepper

**Fig. 7:**
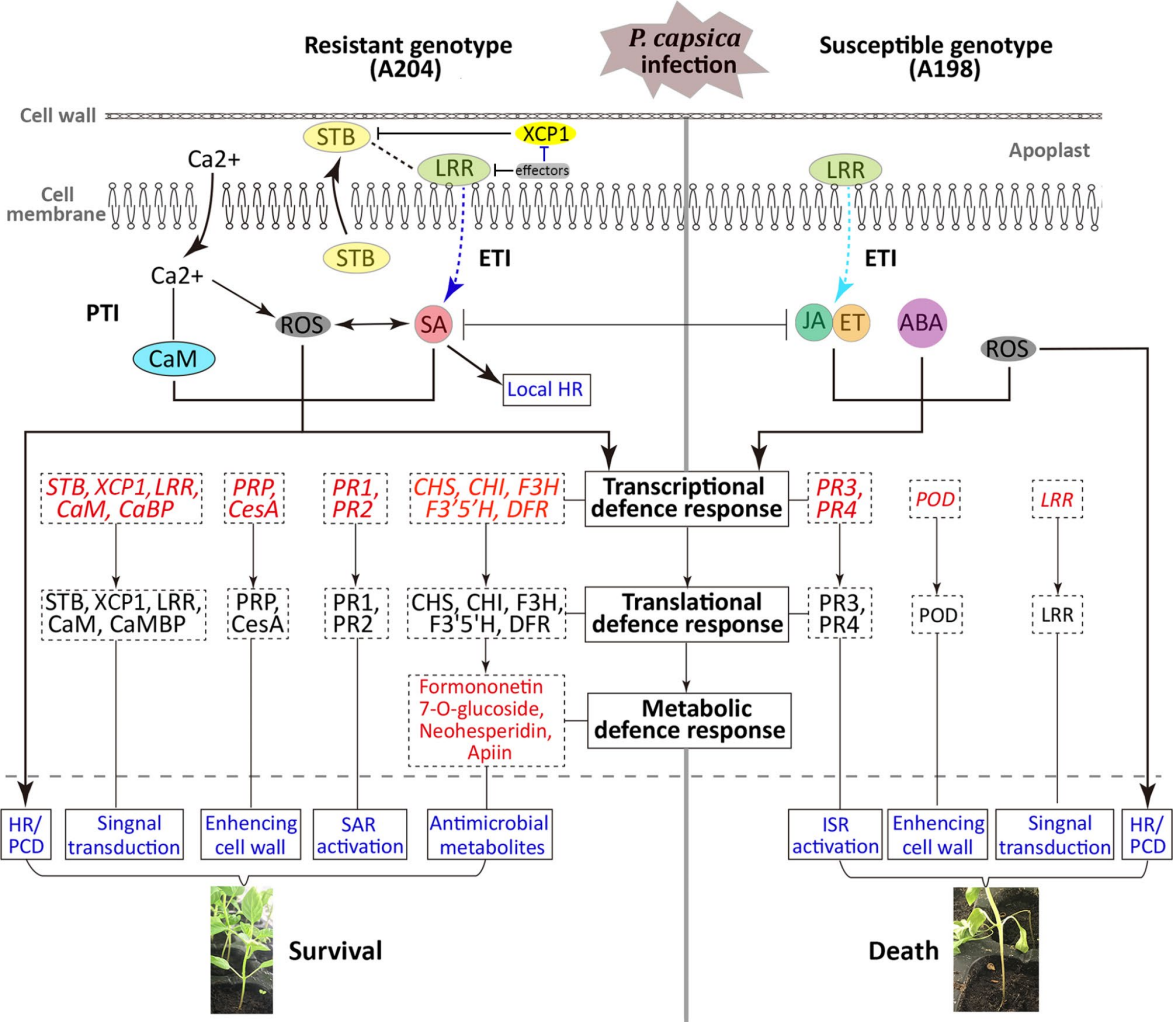
Proposed model for pepper (A204 and A198) roots in response to *P. capsici* infection. The blue and cyan dotted arrows represent strong and weak signal transduction, respectively. ABA, abscisic acid; CaM, calmodulin; CesA, cellulose synthase; CHI, chalcone isomerase; CHS, chalcone synthase; DFR, dihydroflavonol reductase; ET, ethylene; ETI, effector-triggered immunity; F3H, flavanone 3-hydroxylase; F3’5’H, flavonoid 3’,5’-hydroxylase; HR, hypersensitive response; ISR, induced systemic resistance; JA, jasmonic acid; LRR, leucine-rich repeat; PCD, programmed cell death; POD, peroxidase; PRP, proline-rich protein; PR1, pathogenesis-related protein 1; PR2, endo-1,3(4)-beta-glucanase; PR3 and PR4, chitinase; PTI, pattern-recognition receptor (PRR)-triggered immunity; ROS, reactive oxygen species; SA, salicylic acid; SAR, systemic acquired resistance; SBT, subtilisin-like proteases; XCP1, xylem cysteine proteinase 1

Based on the functions in the resistance of the DEGs and DAMs and their expression patterns suggested by comparative transcriptome and metabolome data, this study proposed a possible mechanism for the cellular response to *P*. *capsici* in pepper roots (Fig. 7). For the resistant genotype, *P*. *capsici* infection was quickly perceived by the SBTs and/ or XCP1 in the apoplast. This perception promoted the flow of Ca^2+^ into the cytoplasm to activate CaMs and ROS and to induce a primary immune response, including HR/PCD, indicating the activation of PTI. In addition, the information of the perception was transformed by LRRs on the surface of the cell membrane to transmit signals to the cytoplasm and activate the downstream SA signaling pathway, suggesting that ETI was triggered as the second line of defense. Furthermore, ROS activated downstream signaling cascades, as well as SA signaling, to induce overall transcriptional reprogramming, favoring defense. First, the expression of *PR1* and *PR2* indicated the activation of the SA-mediated SAR; second, the upregulated genes, *PRP* and *CesA*, were related to cell wall reinforcement to enhance the structural resistance; third, the activated flavonoid biosynthesis pathways (upregulated *CHS*, *CHI*, *F3H*, *F3’5’H*, and *DFR*) increased the accumulation of antibacterial secondary metabolites (neohesperidin, apiin, and formononetin 7-O-glucoside) to defend against *P*. *capsici*; and fourth, the activation of genes (*SBT*, *XCP1*, *LRR*, *CaM*, and *CaMBP*) associated with pathogen recognition and signaling ensured a sustained and efficient defense response. Ultimately, the plants survive the pathogen through timely signaling and an effective defense response. For the susceptible genotype, LRR-related genes were highly expressed at 48 hpi, indicating delayed or abnormal ETI due to weakened PTI, which is an indispensable component of ETI during bacterial infection [61]. Although susceptible pepper plants attempted to resist *P*. *capsici* by activating JA/ET-mediated ISR and employing peroxidase, which was involved in cell wall enhancement, this failed to resist the continuous attack of *P*. *capsici* and repair the damage caused by *P*. *capsici*, which ultimately resulted in the spread of the disease and the death of the plant. Our results not only help us to obtain a deeper understanding of the defense response of plants to pathogens, but also have potential in guiding *P. capsici*-resistant genetic engineering.

## Conclusions

In summary, the whole transcriptome and metabolome of pepper roots infected by *P*. *capsici* at 24 and 48 hpi were characterized using RNA-seq and UPLC-MS/MS, respectively. More DEGs and DAMs in the resistant genotype A204 at the early stage of infection indicated the prompt activation of defense responses in A204. Interestingly, genes encoding two proteases involved in pathogen recognition, SBTs and XCP1, were induced in A204, which may cause timely initiation of the defensive response and normal PTI in the resistant genotype. Furthermore, in the resistant genotype, Ca^2+^- and SA-mediated signaling pathways induced overall transcriptional reprogramming and activated different defense mechanisms, such as cell wall reinforcement (*PRP* and *CesA*) and the activation of SAR (*PR1* and *PR2*) and flavonoid biosynthesis pathways (*CHS*, *CHI*, *F3H*, *F3’5’H*, and *DFR*). In the susceptible genotype, the weakened PTI resulted in delayed ETI, which activated downstream JA/ET-mediated ISR (*PR3* and *PR4*) to defend against *P*. *capsici*. Although DEG functional analysis is required to further understand the roles of these DEGs in symptom formation, the potential candidate DEGs may provide a starting point in the elucidation of the molecular mechanism underlying resistance characteristics and may provide potential genetic resources for the improvement of *P*. *capsici* resistance characteristics in pepper.

## Materials and methods

### Plant materials, growth conditions, and inoculations

Two pepper accessions with obvious differences in resistance to *P*. *capsici* were obtained by screening 200 pepper germplasms for *P*. *capsici* resistance [62]. Resistant genotype A204 (called R), a landrace collected from south China, is highly resistant to *P*. *capsici*, similar to the resistant material CM334. Susceptible genotype A198 (called S) from north China shows obvious susceptibility to all *P*. *capsici* isolates in Jiangxi Province, China. Seeds were sown in 32-cell plastic trays filled with growth substrate (Pindstrup, Ryomgaard, Denmark), and seedlings were cultivated in a phytotron FITOCLIMA 10,000 PLH (Aralab, Sintra, Portugal) at 28.0 ±0.5 °C/ 25.0 ± 0.5 °C (14 h day/10 h night), and 70% humidity. *Phytophthora capsici* isolate Ga1 (race 2) used in this study was isolated from infected pepper plants in a pepper production field in 2020 in Gaoan, Yichun, Jiangxi Province, China (E 115°18′, N 28°20′). Plants at the four-to-six true leaf stage were inoculated with *P*. *capsici* isolate Ga1 with following method. In brief, 12 h before inoculation, seedlings were watered well, and 3 mL zoospore suspension with a concentration of 1 × 10^4^ zoospores/mL was poured on the growth substrate surface of each cell the next morning. The inoculated plants were kept at 26 ± 1 °C and 80% relative humidity in the day and night cycle, and visible lesions on the base of the stem (shoot–root junction) were recorded every 12 h after inoculation.

The whole roots were sampled at 0, 24, and 48 hpi. For each genotype, roots from four plants at each time point were harvested, rinsed three times with distilled water, packed in aluminum foil, and snap-frozen in liquid nitrogen until further usage. A total of 36 pepper root samples were harvested, with six biological replicates for each genotype at each time point (6 × 2 × 3). Among them, the first three replicates at each time point for each genotype were used for transcriptome and metabolome analysis, and the others were used only for metabolome analysis.

### RNA extraction, cDNA library preparation, and sequencing

Total RNA was isolated from 18 pepper roots using the RNeasy Plus Plant Kit (Qiagen, Hilden, Germany), and the genomic DNA was removed using the RNeasy®MinElute® Cleanup Kit (Qiagen, Hilden, Germany) following the manufacturer’s instructions. The RNA concentration and purity were measured using NanoDrop 2000 (Thermo Fisher Scientific, Wilmington, DE, USA). The RNA integrity (RIN > 8.0) was assessed using the RNA Nano 6000 Assay Kit of the Agilent Bioanalyzer 2100 system (Agilent Technologies, Palo Alto, CA, USA). Sequencing libraries were then prepared using the NEBNext®Ultra™ RNA Library Prep Kit for Illumina (NEB, Ipswich, MA, USA) following the manufacturer’s protocols, and index codes were added to attribute sequences to each sample. Briefly, mRNA was purified from 1 µg total RNA per sample using poly-T oligo-attached magnetic beads. Subsequently, the purified RNA was broken into short fragments using NEBNext First Strand Synthesis Reaction Buffer, and the short fragment RNA was used as a template to synthesize the first-strand cDNA with random hexamer primers and M-MuLV Reverse Transcriptase. Second-strand cDNA synthesis was subsequently performed using DNA Polymerase I and RNase H. After the adenylation of the 3’ ends of the DNA fragments, the NEBNext Adaptor with hairpin loop structure was ligated to prepare for hybridization. To select cDNA fragments that were preferentially 240 bp in length, the library fragments were purified using the AMPure XP system (Beckman Coulter, Miami, FL, USA). Then, PCR enrichment of adaptor-ligated cDNA was performed with Phusion High-Fidelity DNA polymerase, universal PCR primers, and Index (X) Primer. The PCR products were purified using AMPure XP system (Beckman Coulter, Miami, FL, USA). The library quality was assessed using the Agilent Bioanalyzer 2100 system (Agilent Technologies, Palo Alto, CA, USA). Finally, the clustering of the index-coded samples was performed on a cBot Cluster Generation System (Illumina, San Diego, CA, USA) using the TruSeq PE Cluster Kit v4-cBot-HS (Illumina, San Diego, CA, USA), according to the manufacturer’s instructions. After cluster generation, library preparations were sequenced on an Illumina Novaseq 6000 platform to generate paired-end reads by the Biomarker Biotechnology Corporation (Beijing, China).

### Read assembly and differential expression analysis

Before assembly, raw reads were preprocessed by removing adaptor sequences and low-quality sequences with ‘N’ percentage > 10% and quality scores < Q30 using the Perl program to obtain high-quality reads for downstream analysis. The retained clean reads were mapped to the CM334 reference genome (http://peppergenome.snu.ac.kr/) using HISAT2 (https://daehwankimlab.github.io/hisat2/) and then assembled with StringTie (https://ccb.jhu.edu/software/stringtie/).

For gene expression analysis, clean reads were mapped to assembled sequences to calculate the read counts for each transcript, and then the transcriptional levels of each transcript were estimated and normalized as reads per kilobase of transcript per million mapped reads (RPKM). Differential expression analysis was performed using the R package DEseq2 (https://bioconductor.org/packages/release/bioc/html/DESeq2.html). The threshold false discovery rate (FDR) < 0.01 and log2 fold change (log2 FC) ≥ 1 were used to determine the significantly differentially expressed transcripts. The Pearson coefficient R of the samples was calculated using variance-stabilizing transformed data, while principal component analysis (PCA) was performed on all detected transcripts (log2 values, normalized to RPKM) using Metabo Analyst (version 5.0, https://www.metaboanalyst.ca/).

### Functional annotation and enrichment analysis

The differentially expressed transcripts were annotated based on the NR, Swiss-Prot, Pfam, GO, KEGG, COG, eggNOG, and KOG databases, using BLASTX algorithms with a significant threshold E-value < 1e-5. To optimize the genome annotation information and discover new transcripts and genes, the transcripts (containing exons > 1, coding peptides > 50 amino acids) for which no annotation information was obtained using the above processes were defined as new transcripts. The new transcripts were then annotated based on their amino sequences using HMMER (version 3.3.2, http://www.hmmer.org/) against the Pfam database (E-value < 1e-5). For enrichment analysis, DEGs were assigned to different KEGG pathways (https://www.kegg.jp/kegg/kegg3a.html) using KOBAS (version 3.0, https://www.biostars.org/p/300733/). FDR < 0.05 was considered to indicate reliable enrichment in KEGG pathways.

### RT-qPCR analysis

Twelve DEGs were selected based on their functions and differential expression patterns for RT-qPCR analysis to verify the expression patterns revealed by RNA-seq in both genotypes at each time point. Total RNA extraction and synthesis of first-strand cDNA were performed according to the protocol used for RNA-seq mentioned above. Primers (Supplementary Materials 6: Table S6) were designed using the online primer-BLAST program in NCBI (https://www.ncbi.nlm.nih.gov/tools/primer-blast/). RT-qPCR was performed using the TB Green® Premix Ex Taq™ II (Takara Biomedical Technology Co., Ltd., Beijing, China), following the manufacturer’s protocol on a Bio-Rad CFX96 real-time PCR system. PCR was performed under the following conditions: 95 °C for 30 s, followed by 40 cycles of 95 °C for 5 s and 60 °C for 30 s. The detection threshold cycle for each reaction was normalized against the expressed level of the pepper reference gene *UBI-3* (GenBank ID: AY486137) [63]. Three technical replicates were performed for each target gene, and the 2^-ΔΔCt^ method [64] was used to determine the relative expression of all DEGs. Three biological replicates were performed for the RT-qPCR experiment.

### Untargeted metabolome detection

The freeze-dried root samples were pulverized using a mortar and pestle. A total of 100 mg of pulverized samples was taken and placed in an Eppendorf tube and extracted with 300 µL of methanol containing 20 µL internal standard substances (L-2-chlorophenylalanine). The samples were homogenized for 30 s and then treated with ultrasound for 10 min in an ice water bath. After incubation at − 20 °C for 1 h to precipitate the proteins, the samples were centrifuged at 11,000 g for 15 min at 4 °C (Thermo Fisher Scientific Heraeus Fresco 21, Waltham, MA, USA), and 200 µL of the supernatant was transferred into a fresh 2 mL LC/MS glass vial. To ensure the stability and reliability of the data generated using liquid chromatography–tandem mass spectrometry (LC-MS/MS), a quality control sample was prepared by pooling 20 µL of the above supernatant from each sample.

The separation of compounds was conducted using an Agilent 1290 infinity UPLC system (Agilent Technologies, Palo Alto, CA, USA) equipped with an ACQUITY UPLC BEH Amide column (1.7 μm, 2.1*100 mm) (Waters, Milford, MA, USA). For UPLC separation, 3 µL of each sample was eluted using the mobile phase consisting of solvent A (25 mM NH_4_OAc and 25 mM NH_4_OH in water, pH = 9.75) and solvent B (acetonitrile), with a 0.5 mL min^− 1^ flow rate. The elution gradient was performed as follows: 0–0.5 min (5% A, 95% B), 0.5–7 min (5–35% A, 95–65% A),7–8 min (35–60% A, 65–40% A), 8–9 min (60% A, 40% B), 9–9.1 min (60–5% A, 40–95% B), and 9.1–12 min (95% B, 5% A). The separated compounds were then identified using a high-resolution tandem mass spectrometry (MS/MS) instrument, TripleTOF 5600 (AB Sciex, Framingham, MA, USA), and the acquisition of MS/MS spectra was conducted on an information-dependent basis (IDA) during the LC-MS/MS experiment. In this mode, the acquisition software (Analyst TF 1.7, AB Sciex) continuously evaluated the full scan survey MS data as it collected and triggered the acquisition of MS/MS spectra depending on preselected criteria. The cycle time was 0.56 s, and during each cycle, 12 precursor ions with an intensity greater than 100 were chosen for fragmentation at a collision energy of 30 eV. The ESI source conditions were set as follows: ion source gas 1 at 60 Psi, gas 2 at 60 Psi, curtain gas at 35 Psi, source temperature 650 °C, and ion spray voltage floating 5000 V or − 4000 V in positive or negative modes, respectively.

### Metabolomics data analysis

The proprietary raw data format generated using the MS/MS instrument was converted to the open mzXML format using ProteoWizard (http://www.proteowizard.org/). Following data conversion, the data were processed using a custom graphical user interface in R package XCMS (version 3.2, https://github.com/sneumann/xcms) for operations, including retention time (RT) correction, peak identification, peak extraction, peak integration, and peak alignment. MINFRAC was set to 0.5, and the cutoff was set to 0.6. The abundance (peak intensity) of the compounds was normalized using the peak area normalization method; that is, each metabolite in each sample was divided by the total peak area in that sample. After processing, a data matrix consisting of the RT, the mass-to-charge ratio (*m/z*) values, and the peak intensity was generated. Peak annotation was then performed using the R package CAMERA (version 1.28.0, https://www.rdocumentation.org/packages/CAMERA/versions/1.28.0). PCA was then performed using the pcaMethods package in the R statistical program (http://www.bioconductor.org/packages/release/bioc/html/pcaMethods.html), and partial least squares-discriminant analysis (PLS-DA) was performed to maximize metabolome differences between the control and treatment samples and obtain variable importance in projection (VIP) values.

To screen differentially accumulated compounds, the data matrix was subjected to a Student’s *t*-test and a fold change (FC) analysis in R, and DAMs were defined according to the following criteria: *p*-value < 0.05, VIP > 1, and FC > 2 or < 0.5. For DAM identification, the accurate masses and isotopic peaks were first searched in the Metlin and KEGG databases (https://www.kegg.jp/kegg/kegg1.html), and then MS/MS spectra were searched in the Metlin database and an in-house MS2 database, according to the Metabolomics Standards Initiative (MSI) of the Chemical Analysis Working Group (CAWG). Finally, the confirmed DAMs were classified according to the KEGG and PubChem compound database (https://www.ncbi.nlm.nih.gov/pccompound), and their pathways were retrieved from KEGG (https://www.kegg.jp/kegg/kegg3.html) and PlantCyc Pathways (Version 9.0, http://pathway.gramene.org/plantcyc.html).

### Electronic supplementary material

Below is the link to the electronic supplementary material.

Supplementary Material 1

Supplementary Material 2

Supplementary Material 3

Supplementary Material 4

Supplementary Material 5

Supplementary Material 6

Supplementary Material 7

## Data Availability

The data of RNA-sequencing is available at National Center for Biotechnology Information (NCBI) with accession number PRJNA960669.

## List of abbreviations

ABA: abscisic acid
CaM: calmodulin
CaMBP: calmodulin-binding protein
CesA: cellulose synthase
CHI: chalcone isomerase
CHS: chalcone synthase
COG: Cluster of Orthologous Group
DFR: dihydroflavonol reductase
DAMs: differentially accumulated metabolites
DEGs: differentially expressed genes
ETI: effector-triggered immunity
eggnog: evolutionary genealogy of genes: Non-supervised Orthologous Groups
ET: ethylene
FDR: false discovery rate
F3H: flavanone 3-hydroxylase
F3’5'H: flavonoid 3’,5’-hydroxylase
FLS: flavonol synthase
FG2: flavonol-3-O-glucoside L-rhamnosyltransferase
FC: fold change
GO: Gene Ontology
hpi: hours post-inoculation
HR: hypersensitive response
ISR: induced systemic resistance
JA: jasmonic acid
KOG: Karyotic Ortholog Groups
LRR: leucine-rich repeat
LOX: lipoxygenase
*m/z*: mass-to-charge ratio
MAPK: mitogen-activated protein kinase
NR: non-redundant
OPLS-DA: orthogonal partial least squares-discriminant analysis
PLCPs: papain-like cysteine proteases
PLS-DA: partial least squares-discriminant analysis
PAMP: pathogen-associated molecular pattern
PR: pathogenesis-related protein
PTI: PAMP-triggered immunity
PE: pectinesterase
PG: polygalacturonase
PCs: principal components
PCA: principal component analysis
PCD: programmed cell death
PRP: proline-rich protein
ROS: reactive oxygen species
RPKM: reads per kilobase of transcript per million mapped reads
RT: retention time
SA: salicylic acid
SBTs: subtilisin-like proteases
SAR: systemic acquired resistance
UPLC-MS/MS: ultra-high performance liquid chromatography and tandem mass spectrometry
VIP: variable importance in projection
VR: vestitone reductase
XCP1: xylem cysteine proteinase 1
