# Supplementary material for: Transcriptome and metabolome analyses revealed the response mechanism of pepper roots to Phytophthora capsici infection
Source: BMC Genomics. 2023 Oct 20;24:626. doi: 10.1186/s12864-023-09713-7 (PMC10589972; doi:10.1186/s12864-023-09713-7)
Supplement: Supplementary file 7 — Supplementary Material 7 [file 12864_2023_9713_MOESM7_ESM.pdf]

Transcriptome and Metabolome Analyses Revealed the Response Mechanism of Pepper Roots to *Phytophthora capsici* Infection

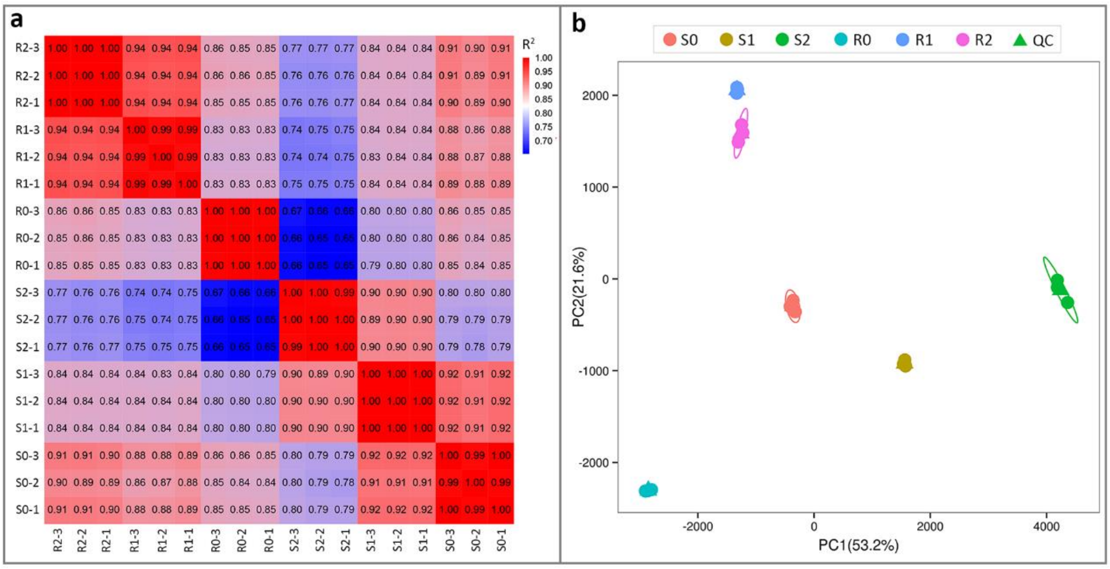

Fig. S1 Corralation and principal component analysis (PCA) of all samples. (**a**) Pearson correlation among 18 samples of two genotypes at three time points. R2: square of the Pearson correlation coefficient. R0-1, R0-2, and R0-3 (S0-1, S0-2, and S0-3) represent three biological replicates of the resistant genotype A204 (susceptible genotype A198) at 0 hpi; R1-1, R1-2, and R1-3 (S1-1, S1-2, and S1-3) represent three biological replicates of the resistant genotype A204 (susceptible genotype A198) at 24 hpi; R2-1, R2-2, and R2-3 (S2-1, S2-2, and S2-3) represent three biological replicates of the resistant genotype A204 (susceptible genotype A198) at 48 hpi. (**b**) PCA of all transcripts of two genotypes at three time points.

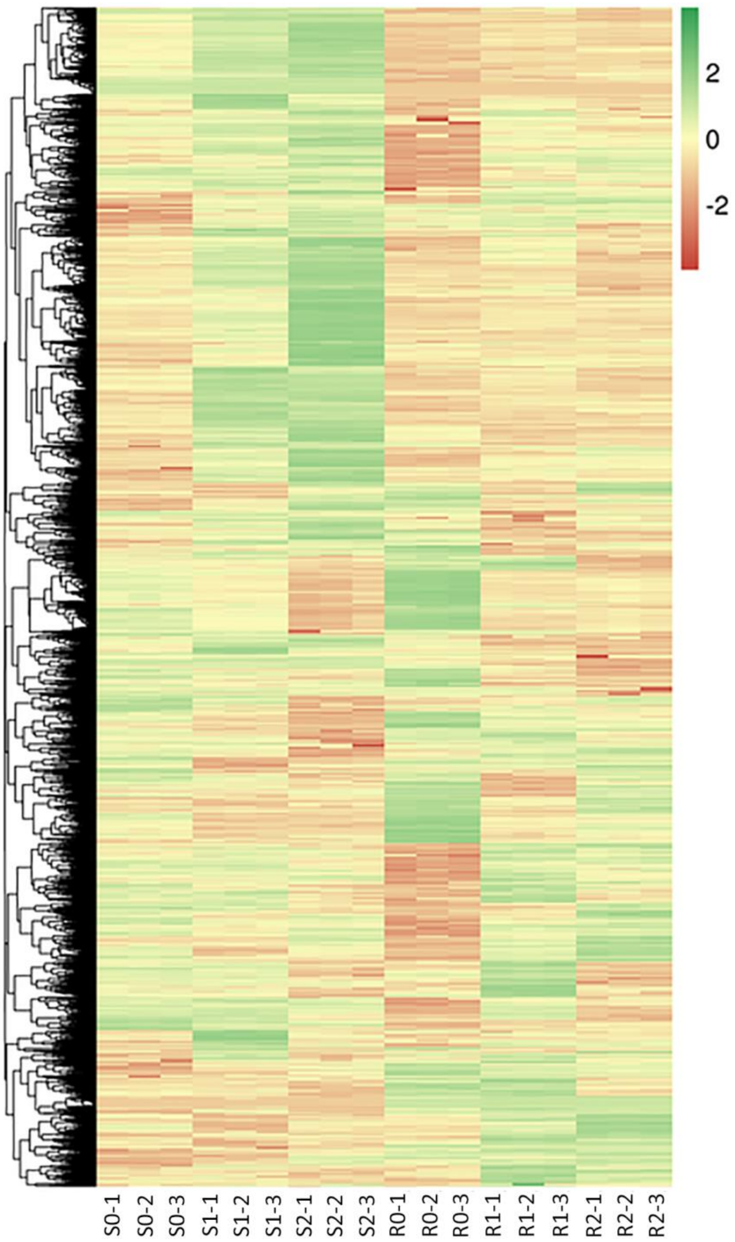

Fig. S2 Cluster analysis for the expressed genes in the A204 and A198. S0-1, S0-2, and S0-3 indicate three duplicates of the susceptible genotype A198 at 0 hours post-inoculation (hpi); S1-1, S1-2, and S1-3 indicate three duplicates of the susceptible genotype A198 at 24 hpi; S2-1, S2-2, and S2-3 indicate three duplicates of the susceptible genotype A198 at 48 hpi; R0-1, R0-2, and R0-3 indicate three duplicates of the resistant genotype A204 at 0 hpi; R1-1, R1-2, and R1-3 indicate three duplicates of the resistant genotype A204 at 24 hpi; R2-1, R2-2, and R2-3 indicate three duplicates of the resistant genotype A204 at 48 hpi

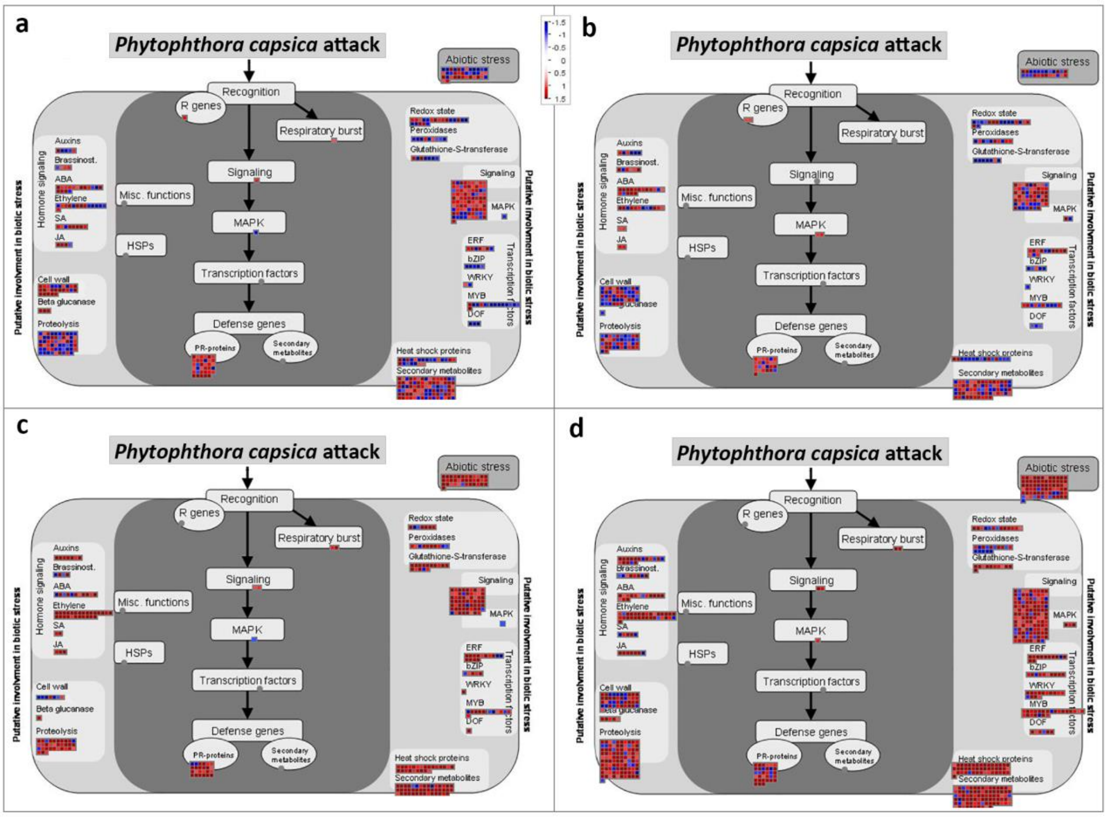

Fig. S3 MapMan overview (Biotic stress panel) of the response of pepper roots to *P. capsica* infection. Expression changes of genes in resistant A204 at 24 hpi (**a**) and 48 hpi (**c**), and susceptible A198 at 24 hpi (b) and 48 hpi (**d**). The colour scale indicates the log2 FC expression values. Up-regulated and down-regulated transcripts are shown in red and blue, respectively.

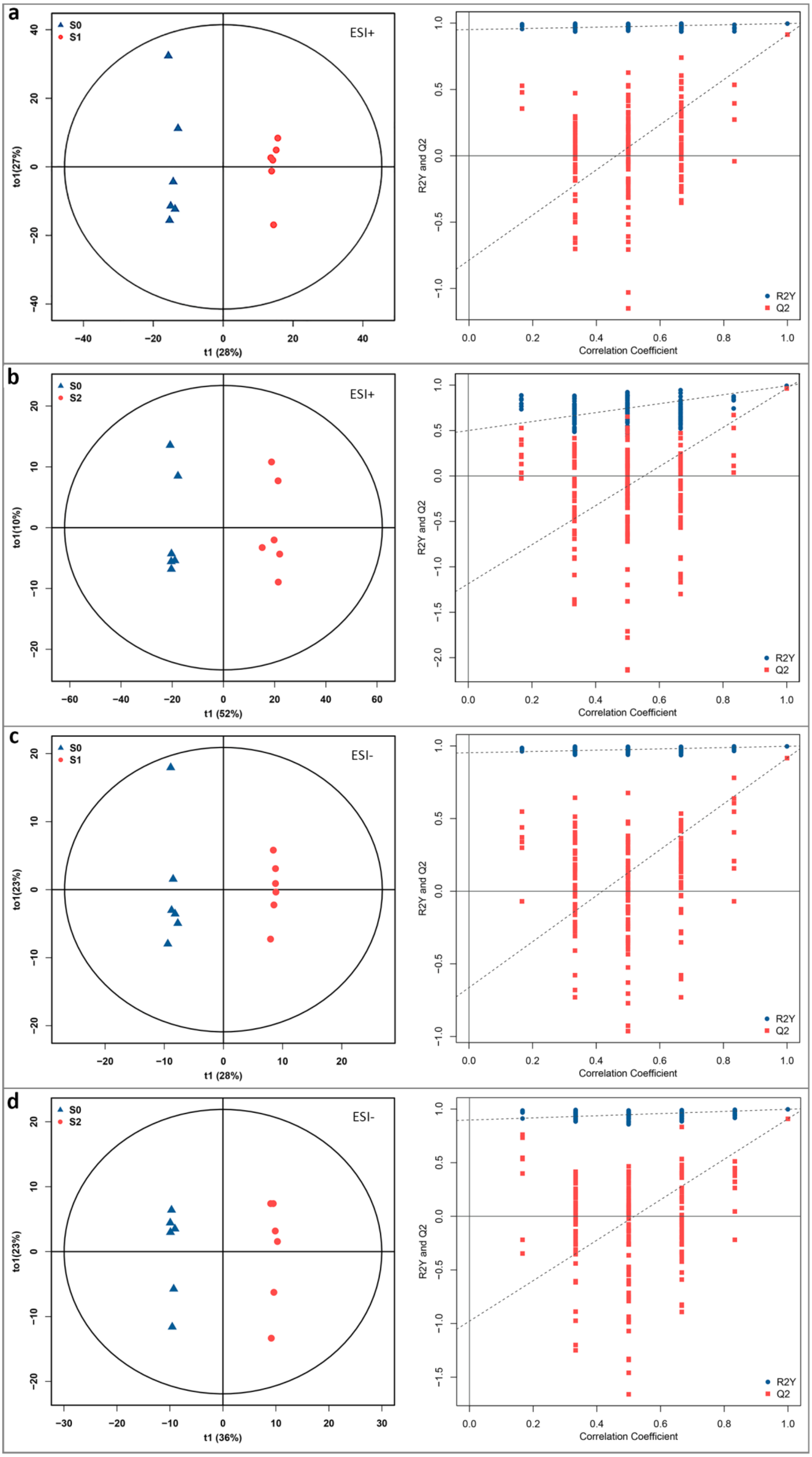

a
S0
S1
ESI+
to1(27%)
t1 (28%)
R2Y and Q2
Correlation Coefficient
R2Y
Q2
b
S0
S2
ESI+
to1(10%)
t1 (52%)
R2Y and Q2
Correlation Coefficient
R2Y
Q2
c
S0
S1
ESI-
to1(23%)
t1 (28%)
R2Y and Q2
Correlation Coefficient
R2Y
Q2
d
S0
S2
ESI-
to1(23%)
t1 (36%)
R2Y and Q2
Correlation Coefficient
R2Y
Q2

Fig. S4 Score scatter plot and permutation test of Orthogonal Partial Least Squares-Discriminant Analysis (OPLS-DA) model for metabolites in susceptible pepper plant roots. (**a**) and (**b**) indicate S1 vs. S0 and S2 vs. S0 in positive ion mode (ESI+), respectively; (**c**) and (**d**) indicate S1 vs. S0 and S2 vs. S0 in negative ion mode (ESI-), respectively.

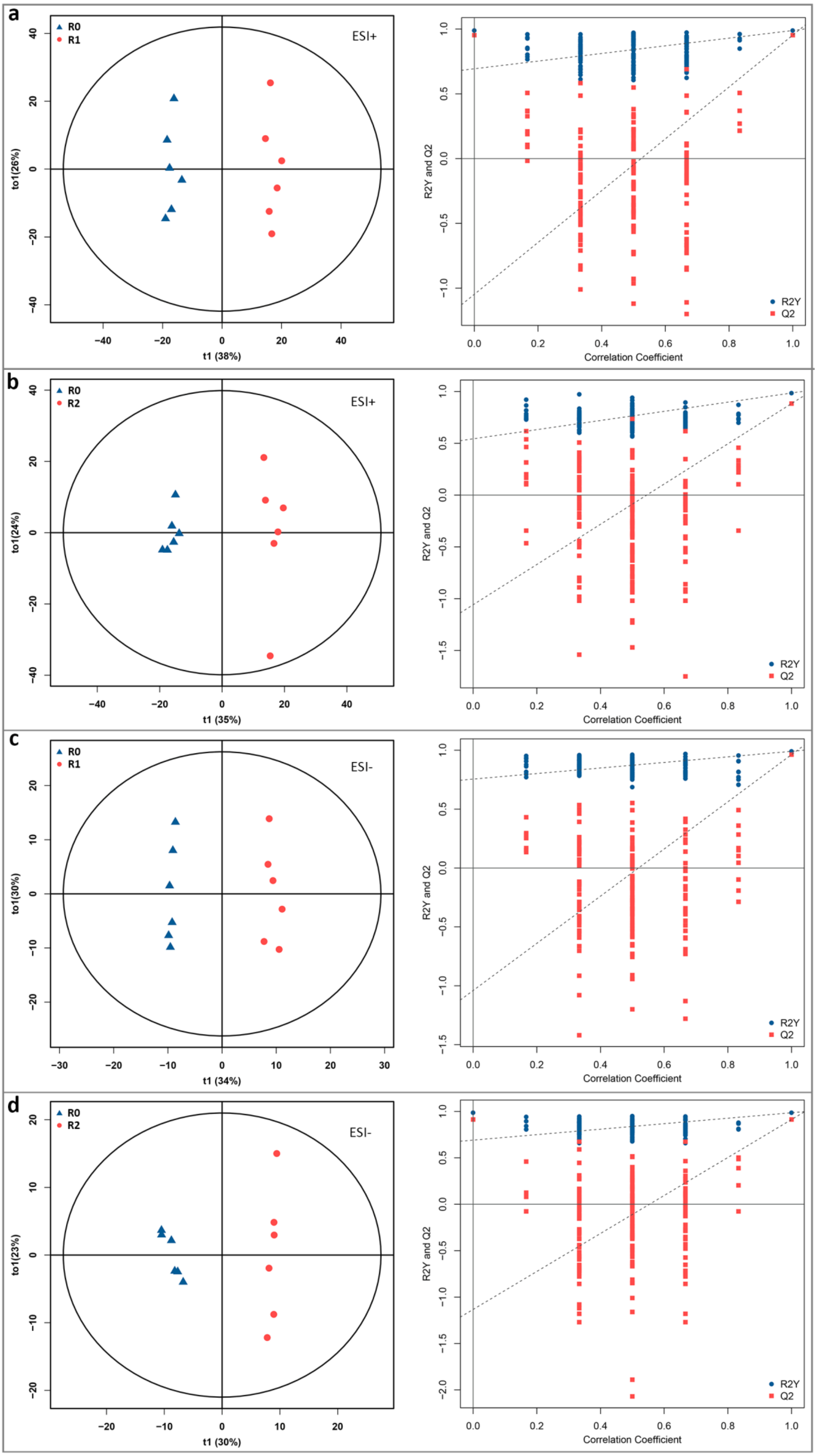
a
R0
R1
ESI+
to1 (26%)
t1 (38%)
R2Y and Q2
Correlation Coefficient
R2Y
Q2
b
R0
R2
ESI+
to1 (24%)
t1 (35%)
R2Y and Q2
Correlation Coefficient
R2Y
Q2
c
R0
R1
ESI-
to1 (30%)
t1 (34%)
R2Y and Q2
Correlation Coefficient
R2Y
Q2
d
R0
R2
ESI-
to1 (23%)
t1 (30%)
R2Y and Q2
Correlation Coefficient
R2Y
Q2

Fig. S5 Score scatter plot and permutation test of Orthogonal Partial Least Squares-Discriminant Analysis (OPLS-DA) model for metabolites in resistant pepper plant roots. (**a**) and (**b**) indicate S1 vs. S0 and S2 vs. S0 in positive ion mode (ESI+), respectively; (**c**) and (**d**) indicate S1 vs. S0 and S2 vs. S0 in negative ion mode (ESI-), respectively.
